# 4-Bromo-*N*-phenyl­benzamide

**DOI:** 10.1107/S1600536812013487

**Published:** 2012-03-31

**Authors:** Hoong-Kun Fun, Suchada Chantrapromma, Weerawat Sripet, Pumsak Ruanwas, Nawong Boonnak

**Affiliations:** a X-ray Crystallography Unit, School of Physics, Universiti Sains Malaysia, 11800 USM, Penang, Malaysia; b Crystal Materials Research Unit, Department of Chemistry, Faculty of Science, Prince of Songkla University, Hat-Yai, Songkhla 90112, Thailand

## Abstract

The mol­ecule of the title benzamide derivative, C_13_H_10_BrNO, is twisted with the dihedral angle between the phenyl and 4-bromo­phenyl rings being 58.63 (9)°. The central N—C=O plane makes dihedral angles of 30.2 (2) and 29.2 (2)° with the phenyl and 4-bromo­phenyl rings, respectively. In the crystal, mol­ecules are linked by N—H⋯O hydrogen bonds into chains along [100]. C—H⋯π contacts combine with the N—H⋯O hydrogen bonds, to form a three-dimensional network.

## Related literature

For bond-length data, see: Allen *et al.* (1987 ▶). For related structures, see: Johnston & Taylor (2011 ▶); Li & Cui (2011 ▶); Saeed *et al.* (2008 ▶); Sripet *et al.* (2012 ▶). For background to and applications of benzamide derivatives, see: Boonleang & Tanthana (2010 ▶); Brown *et al.* (1991 ▶); Hu *et al.* (2008 ▶); Mobi­nikh­aledi *et al.* (2006 ▶); Olsson *et al.* (2002 ▶); World Health Organization (2003 ▶); Xu *et al.* (2009 ▶). For the stability of the temperature controller used in the data collection, see: Cosier & Glazer (1986 ▶).
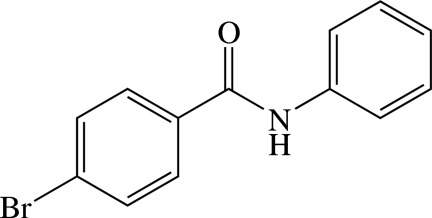


## Experimental

### 

#### Crystal data

C_13_H_10_BrNO
*M* *_r_* = 276.12
Triclinic, 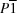

*a* = 5.3552 (2) Å
*b* = 7.6334 (2) Å
*c* = 13.9956 (5) Å
α = 105.757 (3)°
β = 100.585 (3)°
γ = 90.086 (2)°
*V* = 540.45 (3) Å^3^
*Z* = 2
Mo *K*α radiation
μ = 3.78 mm^−1^
*T* = 100 K
0.29 × 0.09 × 0.07 mm

#### Data collection

Bruker APEXII CCD area-detector diffractometer
Absorption correction: multi-scan (*SADABS*; Bruker, 2005 ▶) *T*_min_ = 0.406, *T*_max_ = 0.791
11303 measured reflections
3844 independent reflections
3193 reflections with *I* > 2σ(*I*)
*R*_int_ = 0.032

#### Refinement

*R*[*F* ^2^ > 2σ(*F* ^2^)] = 0.032
*wR*(*F* ^2^) = 0.076
*S* = 1.08
3844 reflections
149 parameters
H atoms treated by a mixture of independent and constrained refinement
Δρ_max_ = 0.66 e Å^−3^
Δρ_min_ = −0.68 e Å^−3^

### 

Data collection: *APEX2* (Bruker, 2005 ▶); cell refinement: *SAINT* (Bruker, 2005 ▶); data reduction: *SAINT*; program(s) used to solve structure: *SHELXTL* (Sheldrick, 2008 ▶); program(s) used to refine structure: *SHELXTL*; molecular graphics: *SHELXTL*; software used to prepare material for publication: *SHELXTL* and *PLATON* (Spek, 2009 ▶).

## Supplementary Material

Crystal structure: contains datablock(s) global, I. DOI: 10.1107/S1600536812013487/sj5220sup1.cif

Structure factors: contains datablock(s) I. DOI: 10.1107/S1600536812013487/sj5220Isup2.hkl

Supplementary material file. DOI: 10.1107/S1600536812013487/sj5220Isup3.cml

Additional supplementary materials:  crystallographic information; 3D view; checkCIF report

## Figures and Tables

**Table 1:** Hydrogen-bond geometry (Å, °) *Cg*1 and *Cg*2 are the centroids of the C1–C6 and C8–C13 rings, respectively.

*D*—H⋯*A*	*D*—H	H⋯*A*	*D*⋯*A*	*D*—H⋯*A*
N1—H1*N*1⋯O1^i^	0.84 (3)	2.37 (3)	3.150 (2)	156 (2)
C2—H2*A*⋯*Cg*2^ii^	0.95	2.77	3.4855 (19)	132
C5—H5*A*⋯*Cg*2^iii^	0.95	2.70	3.4258 (19)	134
C10—H10*A*⋯*Cg*1^iv^	0.95	2.90	3.5444 (19)	126
C13—H13*A*⋯*Cg*1^v^	0.95	2.84	3.4950 (19)	127

Symmetry codes: (i) 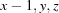; (ii) 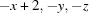; (iii) 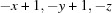; (iv) 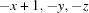; (v) 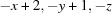.

**Fig. 1.**
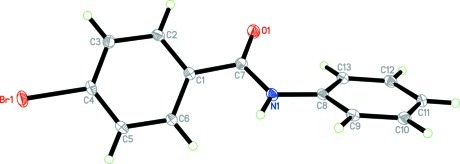
The molecular structure of the title compound, showing 50% probability displacement ellipsoids and the atom-numbering scheme.

**Fig. 2.**
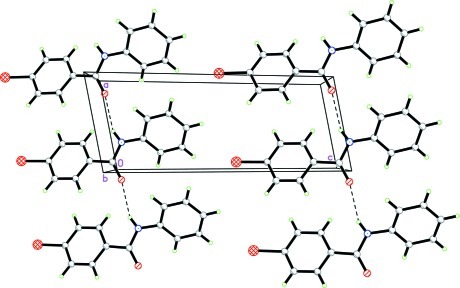
The crystal packing of the title compound viewed along the b axis, showing the molecular chains along the [100] direction. N—H···O hydrogen bonds were drawn as dashed lines.

## supplementary crystallographic information

### Comment

Benzamides are recognised as one of the bioactive skeletons, and some benzamide derivatives exhibit various potent pharmaceutical activities (Brown *et al.*, 1991; Hu *et al.*, 2008). They have been developed as anti-tumor (Olsson *et al.*, 2002), antibacterial (Mobinikhaledi *et al*., 2006) and anti-Alzheimer's agents (Xu *et al.*, 2009). Cisapride (CIS) is an effective benzamide derived drug which can act as a gastrointestinal prokinetic agent. It also has restricted usage for the treatment of gastroesophageal reflux disease in some countries (World Health Organization, 2003) due to its cardiac side effects. The formulation of a more stable CIS oral suspension was studied (Boonleang & Tanthana, 2010). We have synthesized several *N*-phenylbenzamide derivatives in order to evaluate their antibacterial and anti-Alzheimer's activities, and the structure of the title benzamide derivative (I) is reported here.

The molecule of the title benzamide derivative (Fig. 1), C_13_H_10_BrNO, is twisted with the dihedral angle between the phenyl and 4-bromophenyl rings being 58.63 (9)°. The central N-C=O plane is twisted with respect to the two neighbouring ring planes forming dihedral angles of 30.2 (2) and 29.2 (2) ° with the phenyl and 4-bromophenyl rings respectively, and with torsion angles C2–C1–C7–O1 = -28.1 (3)° and C7–N1–C8–C13 = -30.2 (3)°. Bond distances are within normal ranges (Allen *et al.*, 1987) and are comparable to those found in related structures (Johnston & Taylor, 2011; Li & Cui, 2011; Saeed *et al.*, 2008; Sripet *et al.*, 2012).

In the crystal packing (Fig. 2), the molecules are linked by N—H···O hydrogen bonds (Table 1) into chains along the [100] direction. C—H···π contacts involving H atoms from both the phenyl and 4-bromophenyl rings combine with the N—H···O hydrogen bonds to form a 3-dimensional network (Table 1).

### Experimental

To the solution of 4-bromobenzoyl chloride (0.20 g, 0.91 mmol) in acetone (10 ml), aniline (0.12 ml, 1.37 mmol) was added and refluxed for 6 h. After the reaction was completed, a gray solid mass formed which was filtered and washed with distilled water. Colorless needle-shaped single crystals of the title compound suitable for *X*-ray structure determination were recrystallized from acetone/CH_3_OH (1:1 v/v) by slow evaporation of the solvent at room temperature over a week, Mp. 474-475 K.

### Refinement

The amide H atom was located in a difference map and refined isotropically. The aromatic H atoms were positioned geometrically and allowed to ride on their parent atoms, with d(C-H) = 0.95 Å and the *U*_iso_ values were constrained to be 1.2*U*_eq_ of the carrier atom.

### Crystal data

**Table:**

C_13_H_10_BrNO	*Z* = 2
*M**_r_* = 276.12	*F*(000) = 276
Triclinic, *P*1	*D*_x_ = 1.697 Mg m^−^^3^
Hall symbol: -P 1	Melting point = 474–475 K
*a* = 5.3552 (2) Å	Mo *K*α radiation, λ = 0.71073 Å
*b* = 7.6334 (2) Å	Cell parameters from 3844 reflections
*c* = 13.9956 (5) Å	θ = 2.8–32.5°
α = 105.757 (3)°	µ = 3.78 mm^−^^1^
β = 100.585 (3)°	*T* = 100 K
γ = 90.086 (2)°	Needle, colorless
*V* = 540.45 (3) Å^3^	0.29 × 0.09 × 0.07 mm

### Data collection

**Table:**

Bruker APEXII CCD area-detector diffractometer	3844 independent reflections
Radiation source: sealed tube	3193 reflections with *I* > 2σ(*I*)
Graphite monochromator	*R*_int_ = 0.032
φ and ω scans	θ_max_ = 32.5°, θ_min_ = 2.8°
Absorption correction: multi-scan (*SADABS*; Bruker, 2005)	*h* = −7→8
*T*_min_ = 0.406, *T*_max_ = 0.791	*k* = −11→11
11303 measured reflections	*l* = −21→21

### Refinement

**Table:**

Refinement on *F*^2^	Primary atom site location: structure-invariant direct methods
Least-squares matrix: full	Secondary atom site location: difference Fourier map
*R*[*F*^2^ > 2σ(*F*^2^)] = 0.032	Hydrogen site location: inferred from neighbouring sites
*wR*(*F*^2^) = 0.076	H atoms treated by a mixture of independent and constrained refinement
*S* = 1.08	*w* = 1/[σ^2^(*F*_o_^2^) + (0.0335*P*)^2^ + 0.2642*P*] where *P* = (*F*_o_^2^ + 2*F*_c_^2^)/3
3844 reflections	(Δ/σ)_max_ = 0.001
149 parameters	Δρ_max_ = 0.66 e Å^−^^3^
0 restraints	Δρ_min_ = −0.68 e Å^−^^3^

### Special details

**Table:**

Experimental. The crystal was placed in the cold stream of an Oxford Cryosystems Cobra open-flow nitrogen cryostat (Cosier & Glazer, 1986) operating at 120.0 (1) K.
Geometry. All esds (except the esd in the dihedral angle between two l.s. planes) are estimated using the full covariance matrix. The cell esds are taken into account individually in the estimation of esds in distances, angles and torsion angles; correlations between esds in cell parameters are only used when they are defined by crystal symmetry. An approximate (isotropic) treatment of cell esds is used for estimating esds involving l.s. planes.
Refinement. Refinement of F^2^ against ALL reflections. The weighted R-factor wR and goodness of fit S are based on F^2^, conventional R-factors R are based on F, with F set to zero for negative F^2^. The threshold expression of F^2^ > 2sigma(F^2^) is used only for calculating R-factors(gt) etc. and is not relevant to the choice of reflections for refinement. R-factors based on F^2^ are statistically about twice as large as those based on F, and R- factors based on ALL data will be even larger.

### Fractional atomic coordinates and isotropic or equivalent isotropic displacement parameters (Å^2^)

**Table:**

	*x*	*y*	*z*	*U*_iso_*/*U*_eq_	
Br1	0.91443 (4)	0.72493 (3)	0.467544 (14)	0.01790 (7)	
O1	1.1259 (3)	0.2407 (2)	0.00304 (11)	0.0175 (3)	
N1	0.6913 (3)	0.2128 (2)	−0.03111 (12)	0.0119 (3)	
H1N1	0.564 (5)	0.226 (4)	−0.003 (2)	0.020 (6)*	
C1	0.9088 (4)	0.3807 (2)	0.13585 (13)	0.0112 (3)	
C2	1.1145 (4)	0.3763 (2)	0.21293 (14)	0.0126 (3)	
H2A	1.2533	0.3037	0.1972	0.015*	
C3	1.1178 (4)	0.4768 (2)	0.31221 (14)	0.0134 (3)	
H3A	1.2565	0.4728	0.3646	0.016*	
C4	0.9141 (4)	0.5833 (2)	0.33308 (13)	0.0126 (3)	
C5	0.7083 (4)	0.5916 (2)	0.25806 (14)	0.0130 (3)	
H5A	0.5720	0.6666	0.2739	0.016*	
C6	0.7057 (4)	0.4879 (2)	0.15909 (13)	0.0119 (3)	
H6A	0.5649	0.4902	0.1072	0.014*	
C7	0.9210 (4)	0.2720 (2)	0.03006 (14)	0.0128 (3)	
C8	0.6473 (4)	0.1133 (2)	−0.13458 (13)	0.0112 (3)	
C9	0.4262 (4)	0.0002 (2)	−0.17278 (14)	0.0124 (3)	
H9A	0.3161	−0.0131	−0.1289	0.015*	
C10	0.3667 (4)	−0.0932 (2)	−0.27495 (14)	0.0138 (3)	
H10A	0.2160	−0.1701	−0.3005	0.017*	
C11	0.5252 (4)	−0.0749 (2)	−0.33980 (13)	0.0138 (3)	
H11A	0.4832	−0.1381	−0.4097	0.017*	
C12	0.7467 (4)	0.0368 (3)	−0.30173 (14)	0.0141 (3)	
H12A	0.8567	0.0487	−0.3459	0.017*	
C13	0.8091 (4)	0.1317 (2)	−0.19935 (14)	0.0125 (3)	
H13A	0.9603	0.2080	−0.1740	0.015*	

### Atomic displacement parameters (Å^2^)

**Table:**

	*U*^11^	*U*^22^	*U*^33^	*U*^12^	*U*^13^	*U*^23^
Br1	0.02581 (12)	0.01545 (9)	0.01116 (9)	0.00385 (7)	0.00550 (7)	0.00021 (6)
O1	0.0126 (7)	0.0236 (7)	0.0141 (6)	0.0029 (6)	0.0038 (5)	0.0005 (5)
N1	0.0110 (7)	0.0129 (7)	0.0117 (7)	0.0007 (6)	0.0045 (6)	0.0016 (5)
C1	0.0114 (8)	0.0100 (7)	0.0121 (7)	−0.0008 (6)	0.0039 (6)	0.0020 (6)
C2	0.0107 (8)	0.0118 (7)	0.0148 (8)	0.0018 (6)	0.0033 (6)	0.0021 (6)
C3	0.0147 (9)	0.0133 (8)	0.0119 (8)	0.0007 (7)	0.0021 (6)	0.0033 (6)
C4	0.0171 (9)	0.0097 (7)	0.0110 (7)	−0.0002 (7)	0.0057 (6)	0.0010 (6)
C5	0.0142 (9)	0.0108 (7)	0.0148 (8)	0.0022 (7)	0.0063 (7)	0.0027 (6)
C6	0.0125 (8)	0.0111 (7)	0.0116 (7)	0.0014 (6)	0.0026 (6)	0.0021 (6)
C7	0.0136 (9)	0.0127 (8)	0.0121 (8)	0.0021 (7)	0.0029 (6)	0.0034 (6)
C8	0.0135 (8)	0.0090 (7)	0.0115 (7)	0.0028 (6)	0.0031 (6)	0.0031 (6)
C9	0.0115 (8)	0.0109 (7)	0.0154 (8)	0.0017 (6)	0.0042 (6)	0.0037 (6)
C10	0.0116 (9)	0.0129 (8)	0.0159 (8)	0.0007 (7)	0.0019 (7)	0.0030 (6)
C11	0.0148 (9)	0.0131 (8)	0.0109 (7)	0.0027 (7)	0.0015 (6)	−0.0004 (6)
C12	0.0154 (9)	0.0148 (8)	0.0137 (8)	0.0037 (7)	0.0060 (7)	0.0045 (6)
C13	0.0124 (9)	0.0116 (7)	0.0140 (8)	0.0015 (7)	0.0040 (6)	0.0033 (6)

### Geometric parameters (Å, º)

**Table:**

Br1—C4	1.8985 (18)	C5—H5A	0.9500
O1—C7	1.228 (2)	C6—H6A	0.9500
N1—C7	1.361 (2)	C8—C9	1.395 (3)
N1—C8	1.417 (2)	C8—C13	1.396 (3)
N1—H1N1	0.84 (3)	C9—C10	1.390 (3)
C1—C6	1.395 (3)	C9—H9A	0.9500
C1—C2	1.401 (2)	C10—C11	1.384 (3)
C1—C7	1.501 (2)	C10—H10A	0.9500
C2—C3	1.390 (3)	C11—C12	1.391 (3)
C2—H2A	0.9500	C11—H11A	0.9500
C3—C4	1.388 (3)	C12—C13	1.396 (3)
C3—H3A	0.9500	C12—H12A	0.9500
C4—C5	1.390 (3)	C13—H13A	0.9500
C5—C6	1.394 (2)		
			
C7—N1—C8	126.73 (17)	O1—C7—N1	123.92 (17)
C7—N1—H1N1	116.5 (18)	O1—C7—C1	121.13 (17)
C8—N1—H1N1	116.4 (18)	N1—C7—C1	114.94 (16)
C6—C1—C2	119.54 (16)	C9—C8—C13	119.69 (16)
C6—C1—C7	122.67 (16)	C9—C8—N1	117.73 (17)
C2—C1—C7	117.76 (16)	C13—C8—N1	122.50 (17)
C3—C2—C1	120.71 (17)	C10—C9—C8	120.15 (18)
C3—C2—H2A	119.6	C10—C9—H9A	119.9
C1—C2—H2A	119.6	C8—C9—H9A	119.9
C4—C3—C2	118.53 (17)	C11—C10—C9	120.53 (18)
C4—C3—H3A	120.7	C11—C10—H10A	119.7
C2—C3—H3A	120.7	C9—C10—H10A	119.7
C3—C4—C5	122.06 (17)	C10—C11—C12	119.40 (17)
C3—C4—Br1	119.66 (14)	C10—C11—H11A	120.3
C5—C4—Br1	118.28 (14)	C12—C11—H11A	120.3
C4—C5—C6	118.79 (17)	C11—C12—C13	120.79 (18)
C4—C5—H5A	120.6	C11—C12—H12A	119.6
C6—C5—H5A	120.6	C13—C12—H12A	119.6
C5—C6—C1	120.35 (17)	C8—C13—C12	119.43 (17)
C5—C6—H6A	119.8	C8—C13—H13A	120.3
C1—C6—H6A	119.8	C12—C13—H13A	120.3
			
C6—C1—C2—C3	0.2 (3)	C2—C1—C7—O1	−28.1 (3)
C7—C1—C2—C3	178.64 (17)	C6—C1—C7—N1	−29.9 (3)
C1—C2—C3—C4	−0.7 (3)	C2—C1—C7—N1	151.72 (17)
C2—C3—C4—C5	0.1 (3)	C7—N1—C8—C9	153.01 (18)
C2—C3—C4—Br1	−179.15 (14)	C7—N1—C8—C13	−30.2 (3)
C3—C4—C5—C6	0.8 (3)	C13—C8—C9—C10	−0.3 (3)
Br1—C4—C5—C6	−179.88 (14)	N1—C8—C9—C10	176.59 (16)
C4—C5—C6—C1	−1.3 (3)	C8—C9—C10—C11	−0.1 (3)
C2—C1—C6—C5	0.7 (3)	C9—C10—C11—C12	0.5 (3)
C7—C1—C6—C5	−177.57 (17)	C10—C11—C12—C13	−0.6 (3)
C8—N1—C7—O1	−2.4 (3)	C9—C8—C13—C12	0.2 (3)
C8—N1—C7—C1	177.82 (16)	N1—C8—C13—C12	−176.50 (16)
C6—C1—C7—O1	150.22 (19)	C11—C12—C13—C8	0.2 (3)

### Hydrogen-bond geometry (Å, º)

*Cg*1 and *Cg*2 are the centroids of the C1–C6 and C8–C13 rings, respectively.

**Table:**

*D*—H···*A*	*D*—H	H···*A*	*D*···*A*	*D*—H···*A*
N1—H1*N*1···O1^i^	0.84 (3)	2.37 (3)	3.150 (2)	156 (2)
C13—H13*A*···O1	0.95	2.42	2.923 (2)	113
C2—H2*A*···*Cg*2^ii^	0.95	2.77	3.4855 (19)	132
C5—H5*A*···*Cg*2^iii^	0.95	2.70	3.4258 (19)	134
C10—H10*A*···*Cg*1^iv^	0.95	2.90	3.5444 (19)	126
C13—H13*A*···*Cg*1^v^	0.95	2.84	3.4950 (19)	127

Symmetry codes: (i) *x*−1, *y*, *z*; (ii) −*x*+2, −*y*, −*z*; (iii) −*x*+1, −*y*+1, −*z*; (iv) −*x*+1, −*y*, −*z*; (v) −*x*+2, −*y*+1, −*z*.

## References

[bb1] Allen, F. H., Kennard, O., Watson, D. G., Brammer, L., Orpen, A. G. & Taylor, R. (1987). *J. Chem. Soc. Perkin Trans. 2*, pp. S1–19.

[bb2] Boonleang, J. & Tanthana, C. (2010). *Songklanakarin J. Sci. Technol* **32**, 605–611.

[bb3] Brown, J. M., Lemmon, M. J., Horsman, M. R. & Lee, W. W. (1991). *Int. J. Radiat. Biol* **59**, 739–748. 10.1080/09553009114550651 1672362

[bb4] Bruker (2005). *APEX2*, *SAINT* and *SADABS* Bruker AXS Inc., Madison, Wisconsin, USA.

[bb5] Cosier, J. & Glazer, A. M. (1986). *J. Appl. Cryst.* **19**, 105–107.

[bb6] Hu, W.-P., Yu, H.-S., Chen, Y.-R., Tsai, Y.-M., Chen, Y.-K., Liao, C.-C., Chang, L.-S. & Wang, J.-J. (2008). *Bioorg. Med. Chem* **16**, 5295–5302. 10.1016/j.bmc.2008.03.003 18359635

[bb7] Johnston, D. H. & Taylor, C. R. (2011). *Acta Cryst.* E**67**, o2735. 10.1107/S1600536811038062 PMC3201324 22065512

[bb8] Li, H.-L. & Cui, J.-T. (2011). *Acta Cryst.* E**67**, o1596. 10.1107/S1600536811019350 PMC3152108 21837004

[bb9] Mobinikhaledi, A., Forughifar, N., Shariatzadeh, S. M. & Fallah, M. (2006). *Heterocycl. Commun* **12**, 427–430.

[bb10] Olsson, A. R., Lindgren, H., Pero, R. W. & Leanderson, T. (2002). *Br. J. Cancer*, **86**, 971–978. 10.1038/sj.bjc.6600136 PMC2364155 11953831

[bb11] Saeed, A., Hussain, S. & Flörke, U. (2008). *Acta Cryst.* E**64**, o705. 10.1107/S1600536808006430 PMC2961029 21202096

[bb12] Sheldrick, G. M. (2008). *Acta Cryst.* A**64**, 112–122. 10.1107/S0108767307043930 18156677

[bb13] Spek, A. L. (2009). *Acta Cryst.* D**65**, 148–155. 10.1107/S090744490804362X PMC2631630 19171970

[bb14] Sripet, W., Chantrapromma, S., Ruanwas, P. & Fun, H.-K. (2012). *Acta Cryst.* E**68**, o1234. 10.1107/S1600536812010963 PMC3344164 22606167

[bb15] World Health Organization (2003). *Pharmaceuticals: Restriction in Use and Availability, Essential Drugs and Medicines Policy-Quality Assurance and Safely: Medicines Health Technology and Pharmaceuticals* Geneva, Switzerland.

[bb16] Xu, J., Lecanu, L., Tan, M., Greeson, J. & Papadopoulos, V. (2009). *Molecules*, **14**, 3392–3410. 10.3390/molecules14093392 PMC6254727 19783933

